# Repeated endoscopic ultrasound‐guided fine‐needle biopsy of solid pancreatic lesions after previous nondiagnostic or inconclusive sampling

**DOI:** 10.1111/den.14686

**Published:** 2023-10-25

**Authors:** Andrea Lisotti, Anna Cominardi, Maria Cristina Conti Bellocchi, Stefano Francesco Crinò, Alberto Larghi, Antonio Facciorusso, Paolo Giorgio Arcidiacono, Claudio De Angelis, Francesco Maria Di Matteo, Carlo Fabbri, Helga Bertani, Thomas Togliani, Gianenrico Rizzatti, Mario Brancaccio, Antonino Grillo, Alberto Fantin, Alessandro Pezzoli, Francesca D'Errico, Arnaldo Amato, Filippo Antonini, Amedeo Montale, Antonio Pisani, Edoardo Forti, Mauro Manno, Silvia Carrara, Maria Chiara Petrone, Cecilia Binda, Rocco Maurizio Zagari, Pietro Fusaroli, the REUBIO Study Group, Claudio Calvanese, Antonio Maestri, Maurizio Puccetti, Michele Masetti, Stefania Lega, Graziella Masciangelo, Giovanni Aragona, Enrico Gasparini, Giulia Tripodi, Maria Gabriella Pellegrino, Nico Muscatiello, Piera Zaccari, Matteo Tacelli, Livia Archibugi, Giuseppe Vannella, Eleonora Dall'Amico, Serena Stigliano, Gianmarco Marocchi, Chiara Coluccio, Stefano Fabbri, Marianna Signoretti, Santi Mangiafico, Marinella Lupo, Giuseppe Grande, Rita Conigliaro, Emanuele Dabizzi, Alessandro Mussetto, Riccardo Solimando, Rosario Arena, Alberto Merighi, Francesco Decembrino, Giacomo Tamanini, Massimo Oppezzi, Osvaldo Burattini, Rosa Lovero, Massimiliano Mutignani, Tommaso Gabbani, Marco Spadaccini, Alessandro Fugazza, Andrea Anderloni, Benedetto Mangiavillano, Nicole Brighi, Romano Sassatelli, Vincenzo Giorgio Mirante, Paolo Cecinato, Luca Barresi, Donato Alessandro Telesca

**Affiliations:** ^1^ Gastroenterology Unit, Hospital of Imola University of Bologna Imola Italy; ^2^ Gastroenterology Unit Hospital of Piacenza Piacenza Italy; ^3^ Digestive Endoscopy Unit University of Verona Verona Italy; ^4^ Gastroenterology and Digestive Endoscopy Unit University Hospital Borgo Trento Verona Italy; ^5^ Digestive Endoscopy Unit, Fondazione Policlinico Universitario A. Gemelli IRCCS Rome Italy; ^6^ Operative Endoscopy Department, Campus Bio‐Medico University Hospital Rome Italy; ^7^ Gastroenterology Unit, Department of Surgical and Medical Sciences University of Foggia Foggia Italy; ^8^ IRCCS San Raffaele Scientific Institute Vita‐Salute San Raffaele University Milan Italy; ^9^ Digestive Endoscopy Unit ASST Niguarda Milan Italy; ^10^ Endoscopic Unit, Department of Gastroenterology IRCCS Humanitas Research Hospital Milan Italy; ^11^ Department of General and Specialist Medicine, Gastroenterologia‐U Città della Salute e della Scienza di Torino Turin Italy; ^12^ Gastroenterology and Digestive Endoscopy Unit, Forlì‐Cesena Hospitals AUSL Romagna Forli Italy; ^13^ Gastroenterology and Digestive Endoscopy Unit Azienda Ospedaliero Universitaria di Modena Modena Italy; ^14^ Gastroenterology and Digestive Endoscopy Unit Azienda USL Modena Modena Italy; ^15^ Unit of Gastroenterology, Santa Maria delle Croci Hospital AUSL Romagna Ravenna Italy; ^16^ Gastroenterology and Digestive Endoscopy Unit, Rimini “Infermi” Hospital AUSL Romagna Rimini Italy; ^17^ Gastroenterology Unit Veneto Institute of Oncology IOV‐IRCCS Padua Italy; ^18^ Department of Gastroenterology and GI Endoscopy University Hospital Ferrara Italy; ^19^ Gastroenterology and Endoscopy Unit, Ente Ecclesiastico F. Miulli Acquaviva delle Fonti Bari Italy; ^20^ National Institute of Gastroenterology IRCCS Saverio de Bellis Castellana Grotte, Bari Italy; ^21^ Division of Digestive Endoscopy and Gastroenterology Valduce Hospital Como Italy; ^22^ Gastroenterology and Interventional Endoscopy Unit “C. e G. Mazzoni” Hospital Ascoli Piceno Italy; ^23^ Division of Gastroenterology E.O. Galliera Hospital Genoa Italy; ^24^ SSD “Patologie organiche esofago‐gastriche”, IRCCS Azienda Ospedaliero‐Universitaria di Bologna S. Orsola Hospital Bologna Italy; ^25^ Department of Medical and Surgical Sciences – DIMEC University of Bologna Bologna Italy

**Keywords:** fine needle aspiration, neuroendocrine neoplasm, pancreatic cancer, pancreatic ductal adenocarcinoma

## Abstract

**Objectives:**

Repeated endoscopic ultrasound (EUS)‐guided tissue acquisition represents the standard practice for solid pancreatic lesions after previous nondiagnostic or inconclusive results. Since data are lacking, we aimed to evaluate the diagnostic performance of repeated EUS fine‐needle biopsy (rEUS‐FNB) in this setting. The primary outcome was diagnostic accuracy; sample adequacy, sensitivity, specificity, and safety were secondary outcomes.

**Methods:**

Consecutive patients undergoing rEUS‐FNB for solid pancreatic lesions at 23 Italian centers from 2019 to 2021 were retrieved. Pathology on the surgical specimen, malignant histology together with ≥6‐month follow‐up, and benign pathology together with ≥12‐month follow‐up were adopted as gold standards.

**Results:**

Among 462 patients, 56.5% were male, with a median age of 68 (59–75) years, malignancy prevalence 77.0%. Tumor size was 26 (20–35) mm. Second‐generation FNB needles were used in 89.6% cases. Diagnostic accuracy, sensitivity, and specificity of rEUS‐FNB were 89.2%, 91.4%, and 81.7%, respectively (19 false‐negative and 12 false‐positive results). On multivariate analysis, rEUS‐FNB performed at high‐volume centers (odds ratio [OR] 2.12; 95% confidence interval [CI] 1.10–3.17; *P* = 0.03) and tumor size (OR 1.03; 95% CI 1.00–1.06; *P* = 0.05) were independently related to diagnostic accuracy. Sample adequacy was 94.2%. Use of second‐generation FNB needles (OR 5.42; 95% CI 2.30–12.77; *P* < 0.001) and tumor size >23 mm (OR 3.04; 95% CI 1.31–7.06; *P* = 0.009) were independently related to sample adequacy.

**Conclusion:**

Repeated EUS‐FNB allowed optimal diagnostic performance after nondiagnostic or inconclusive results. Patients' referral to high‐volume centers improved diagnostic accuracy. The use of second‐generation FNB needles significantly improved sample adequacy over standard EUS‐FNB needles.

## INTRODUCTION

Pancreatic cancer is the most lethal of neoplasms, with an overall survival dramatically impaired by the difficulty to diagnose patients at early disease stages and a poor response to chemotherapy.^1^

Endoscopic ultrasound (EUS) with tissue acquisition has a crucial role in the detection and characterization of pancreatic neoplasms. EUS‐sampling is indicated not only in patients with unresectable or metastatic neoplasms, but also in patients with localized neoplasms, based on the availability of effective neoadjuvant regimens.^2^, ^3^

However, EUS‐sampling is burdened by a nonnegligible risk of nondiagnostic or inconclusive results, with an estimated negative predictive value not higher than 70–80%.^4^, ^5^ On these premises, in case of nondiagnostic or inconclusive EUS‐sampling, guidelines recommend clinical and radiological follow‐up, re‐evaluation of pathology slides, referral to surgery patients with a high suspicion of malignancy, or repetition of EUS‐guided tissue acquisition.^6^, ^7^, ^8^, ^9^

The diagnostic performance of repeated EUS‐fine‐needle aspiration (rEUS‐FNA) has been validated by a meta‐analysis, including 12 studies with 505 patients.^10^ In this clinical setting, rEUS‐FNA showed a 77% sensitivity and 98% specificity, with a significant contribution of rapid on‐site evaluation (ROSE).

Based on the improved diagnostic performance and the increased worldwide use of fine‐needle biopsy (FNB) needles, repeated EUS‐FNB (rEUS‐FNB) has been hypothesized to be essential after previous inconclusive percutaneous sampling.^11^ Nevertheless, strong data on the diagnostic yield of repeated sampling with FNB needles after nondiagnostic or inconclusive EUS‐guided tissue sampling are lacking.

The aim of this study was to assess the diagnostic performance of rEUS‐FNB in patients with nondiagnostic or inconclusive EUS‐sampling for solid pancreatic lesions. The primary outcome was diagnostic accuracy, while secondary outcomes were rEUS‐FNB sample adequacy, sensitivity, specificity, and incidence of adverse events. Finally, factors related to rEUS‐FNB diagnostic accuracy and sample adequacy were also analyzed.

## METHODS

Detailed description of the material and methods is available in Appendix S1.

### Study design

Thirty Italian centers were invited to a retrospective study retrieving all consecutive patients who underwent rEUS‐FNB for solid pancreatic lesions from January 2019 to December 2021. All adult patients (≥18 years old) who underwent rEUS‐FNB for solid pancreatic lesion characterization after previous nondiagnostic or inconclusive EUS‐guided tissue acquisition were eligible. Cystic neoplasms, previous non‐EUS sampling, repeated tissue sampling different from EUS‐FNB (EUS‐FNA, percutaneous, or surgical), and rEUS‐FNB of extrapancreatic neoplasms were excluded.

The study was first approved in December 2021 by our local Institution Review Board (IRB) (Comitato Etico di Area Vasta Emilia Centro, Italy, protocol number: 978‐2021‐OSS‐AUSLIM‐21185, ID 3369), and subsequently approved by all IRBs of each center. The study protocol was also made available on clinicaltrial.gov (NCT05226572).

All endoscopic procedures were performed by physicians who have completed EUS training and have at least a 2‐year experience and 150 EUS procedures/year; no trainees were involved.

### Gold standard for diagnosis

The gold standard for diagnosis of solid pancreatic lesions was: (i) pathology of the surgical specimen for those who underwent pancreatectomy; (ii) pathology of autoptic examination in case of death, when available; (iii) at least 6‐month assessment of disease evolution through a combination of clinical course, imaging modalities, and/or additional tissue sampling in nonresected patients with proven malignant disease; (iv) at least 12‐month assessment through a combination of clinical course, imaging modalities, and/or additional tissue sampling demonstrating a stable benign condition. The remaining cases were included with sample adequacy analysis, but censored from diagnostic accuracy one.

### Study aims and outcome definitions

The primary end‐point was to assess the diagnostic accuracy of rEUS‐FNB of solid pancreatic lesions. Diagnostic accuracy was defined as the concordance between rEUS‐FNB diagnosis and the gold standard diagnosis. Sample adequacy was defined as the acquisition through rEUS‐FNB of a sufficient specimen to reach a pathology diagnosis.^6^

The secondary end‐points were rEUS‐FNB sample adequacy, diagnostic sensitivity, specificity, and positive and negative predictive values.

Procedure‐related adverse events were defined as immediate or delayed (bleeding, perforation, pancreatitis, or any clinically relevant event) deemed as a consequence of rEUS‐FNB and were graded according to the AGREE classification.^12^, ^13^

End‐cutting FNB needles, such as Franseen type, fork‐tip, forward bevel, and Menghini type needles were included in the second‐generation EUS‐FNB group.

### Statistical analysis

Continuous variables were expressed as mean ± standard deviation (SD) or median (interquartile range, IQR) according to their distribution and compared using Student's *t*‐test or the Mann‐Whitney *U*‐test, respectively. Categorical variables were reported as number and proportion and compared using the χ^2^‐test or Fisher's exact test, when appropriate. Receiver operating characteristic (ROC) curve analysis (Youden's statistic) was used for dichotomization of continuous variables, when necessary.^14^

The logistic regression model was used to identify factors related to rEUS‐FNB sample adequacy and diagnostic accuracy. Variables with a *P*‐value of <0.1 on univariate analysis were included in the multivariable logistic regression model. Odds ratio (OR) together with 95% confidence intervals (CIs) have been reported. Statistical significance was determined as *P* < 0.05 (two‐tailed test). Statistical analyses were performed using MedCalc Statistical Software version 20.110 (MedCalc Software, Ostend, Belgium; https://www.medcalc.org; 2022).

## RESULTS

### Study population

Twenty‐three centers participated; overall, we collected data from 466 cases of rEUS‐FNB of solid pancreatic lesions performed. Of them, four repeat procedures were excluded, since they were performed with an rEUS‐FNA. Finally, 462 rEUS‐FNB after a previous nondiagnostic or inconclusive EUS sampling were included; 261 (56.5%) patients were male, with a median age of 68 (59–75) years. Three hundred and five (66.0%) patients underwent a previous nondiagnostic or inconclusive EUS‐FNB, while 101 (34%) were EUS‐FNA. In 95 (20.6%) cases, previous EUS‐sampling had been performed at a different center; detailed characteristics of previous EUS‐tissue sampling are described in Table S1.

### Pancreatic disease features

In all, 269 (58.2%) lesions were located in the head of pancreas, 45 (9.7%) in the uncinate process, 109 (23.6%) in the body, and 39 (8.4%) in the pancreatic tail. The median lesion size was 26 (20–35) mm. Patient baseline characteristics are summarized in Table 1.

**Table 1:** Characteristics of the study population and procedures

	Total (*n* = 462)
Demographic	
Gender (male), *n* (%)	261 (56.5)
Age (years), median [IQR]	68 [59–75]
Previous EUS‐TA	
Previous EUS‐TA mode	
Previous EUS‐FNA, *n* (%)	157 (34.0)
Previous EUS‐FNB, *n* (%)	305 (66.0)
Solid pancreatic neoplasms	
Location	
Head, *n* (%)	269 (58.2)
Uncinate process, *n* (%)	45 (9.7)
Body, *n* (%)	109 (23.6)
Tail, *n* (%)	39 (8.4)
Size (mm), median [IQR]	26 [20–35]
Size (mm), range	9–150
Size (≤20 mm), *n* (%)	150 (32.5)
Size (≤10 mm), *n* (%)	13 (2.8)
Repeated EUS‐FNB	
At the same center, *n* (%)	367 (79.4)
At another center, *n* (%)	95 (20.6)
High‐volume center, *n* (%)	207 (66.5)
Sedation	
Conscious sedation, *n* (%)	164 (35.5)
Deep sedation, *n* (%)	278 (60.2)
General anesthesia, *n* (%)	20 (4.3)
Puncture route	
Transgastric, *n* (%)	158 (34.2)
Transduodenal, *n* (%)	304 (65.8)
2nd generation EUS‐FNB needle, *n* (%)	414 (89.6)
Franseen type, *n* (%)	220 (47.6)
Fork‐tip, *n* (%)	147 (31.8)
Menghini, *n* (%)	25 (5.4)
Forward bevel, *n* (%)	22 (4.8)
1st generation EUS‐FNB needle, *n* (%)	48 (10.4)
Reverse bevel, *n* (%)	48 (10.4)
EUS‐FNB needle size	
25G, *n* (%)	96 (20.8)
22G, *n* (%)	324 (70.1)
20G, *n* (%)	22 (4.8)
19G, *n* (%)	20 (4.3)
EUS‐FNB needle passes	
1 pass, *n* (%)	44 (9.5)
2 passes, *n* (%)	132 (28.6)
3 passes, *n* (%)	186 (40.3)
4 or more passes, *n* (%)	100 (21.6)
ROSE availability, *n* (%)	112 (24.2)
rEUS‐FNB‐related AEs	
AEs (overall), *n* (%)	15 (3.2)
Mild bleeding, *n*	10
Mild acute pancreatitis, *n*	4
Moderate acute pancreatitis, *n*	1
AEs – AGREE classification, *n* (%)	3 (0.6)
Grade 0 – no AE, *n*	12
Grade I – requiring lab tests, *n*	2
Grade II – requiring hospital admission, *n*	1

AE, adverse event; EUS, endoscopic ultrasound; EUS‐FNA, EUS fine‐needle aspiration; EUS‐FNB, EUS fine‐needle biopsy; EUS‐TA, EUS‐tissue acquisition; IQR, interquartile range; rEUS‐FNB, repeated EUS‐FNB; ROSE, rapid on‐site evaluation.

**Figure 1:**
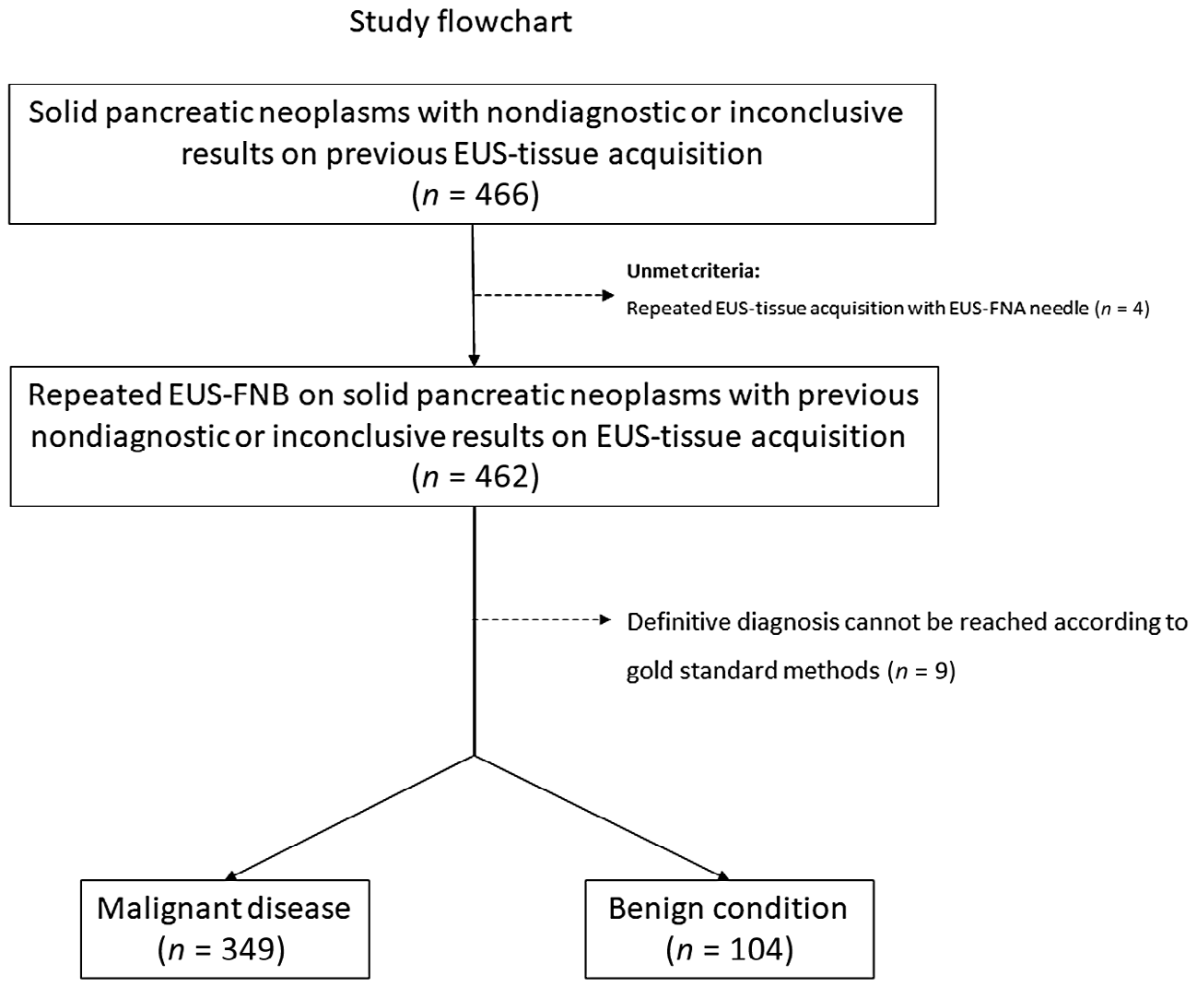
Study flowchart. EUS, endoscopic ultrasound; EUS‐FNA, EUS fine‐needle aspiration; EUS‐FNB, EUS fine‐needle biopsy.

The gold standard for the final diagnosis was pathology on surgical specimen in 119 (25.8%) cases, autopsy in two (0.4%) cases, malignant pathology on tissue sampling together with ≥6‐month of congruent clinical or radiological follow‐up in 268 (58.0%), and benign pathology on tissue sampling together with at least 12‐month clinical and radiological follow‐up in 64 (13.9%). The median follow‐up was 3.5 (2–8) and 14 (12–18) months for malignant and benign cases, respectively. According to the gold standard, 349 (75.5%) patients were ultimately diagnosed with a malignant disease, while 104 (22.5%) had a benign condition. Finally, in nine cases (1.9%) a final diagnosis could not be reached. The study flowchart is shown in Figure 1. The final diagnoses are detailed in Table S2.

### Repeated EUS‐FNB

Most rEUS‐FNB (66.5%) were performed in high‐volume centers, under deep sedation (*n* = 278, 60.2%) or conscious sedation (*n* = 164, 35.5%), while only 20 (4.3%) under general anesthesia with airways intubation. A transduodenal approach was required in 304 (65.8%) cases.

Most rEUS‐FNB were performed using the Franseen type (*n* = 220, 47.6%) and fork‐tip (*n* = 147, 31.8%) needles; the remaining cases with the Menghini type (*n* = 25, 5.4%), reverse bevel (*n* = 48, 10.4%), and forward bevel (*n* = 22, 4.8%) needles. Overall, second‐generation end‐cutting EUS‐FNB needles were used in 414 (89.6%) cases. A 22G needle was used in 324 (70.1%) cases, a 25G needle in 96 (20.8%) cases, a 20G needle in 22 (4.8%), and a 19G in 20 (4.3%). A median of 3 (2, 3) needle passes were performed (Table 1). ROSE was available in 112 (24.2%) cases. Adverse events were described in 15 cases: 10 mild bleeding, four mild acute pancreatitis, and one moderate acute pancreatitis. According to the AGREE classification, most of them (*n* = 12) were grade 0 (no AE), two were grade I requiring lab tests, and one was grade II, requiring hospital admission for 3 days; the incidence of adverse events was 0.6%, accordingly.

### Diagnostic accuracy

The rEUS‐FNB showed an overall diagnostic accuracy of 89.2% (95% CI 86.0–91.9%), with a sensitivity and specificity of 91.4% (95% CI 88.0–94.1%), and 81.7% (95% CI 73.0–88.6%), respectively (Table 2).

**Table 2:** Diagnostic yield of repeated endoscopic ultrasound‐guided fine‐needle biopsy for solid pancreatic lesions

Diagnostic performance	% (95% confidence interval)
Sample adequacy	94.2 (89.2–98.6%)
Malignant disease prevalence	77.0 (72.9–88.6%)
Sensitivity	91.4 (88.0–94.1%)
Specificity	81.7 (73.0–88.6%)
Positive predictive value	94.4 (91.8–96.2%)
Negative predictive value	73.9 (66.5–80.2%)
Diagnostic accuracy	89.2 (86.0–91.9%)
Positive likelihood ratio	5.00 (3.33–7.52)
Negative likelihood ratio	0.11 (0.07–0.15)

Univariate and multivariate analyses of factors related to rEUS‐FNB diagnostic accuracy are reported in Table 3. On univariate analysis, rEUS‐FNB at high‐volume centers (OR 1.62; 95% CI 1.14–2.16; *P* = 0.02) was significantly related to higher diagnostic accuracy. A trend toward higher diagnostic accuracy for previous EUS‐guided tissue acquisition performed at another center (OR 2.33; 95% CI 0.89–6.07; *P* = 0.08), lesion size as a continuous variable in mm (OR 1.03; 95% CI 0.99–1.06; *P* = 0.08), >2 rEUS‐FNB needle passes (OR 1.75; 95% CI 0.95–3.23; *P* = 0.07), and performance of rEUS‐FNB under deep sedation or general anesthesia (OR 1.75; 95% CI 0.95–3.23; *P* = 0.07) were also observed. On the other hand, a trend toward a lower diagnostic accuracy was observed for previous EUS‐guided tissue acquisition performed with EUS‐FNB (OR 0.50; 95% CI 0.24–1.03; *P* = 0.06) and for lesions located in the pancreatic tail (OR 0.48; 95% CI 0.20–1.15; *P* = 0.10).

**Table 3:** Factors related to repeated endoscopic ultrasound‐guided fine‐needle biopsy (rEUS‐FNB) diagnostic accuracy

	Univariate	*P*‐value	Multivariate	*P*‐value
	OR (95% CI)		OR (95% CI)	
Gender (male)	0.72 (0.39–1.36)	0.31	–	–
Age (years)	0.98 (0.95–1.01)	0.11	–	–
Previous EUS‐FNB	0.50 (0.24–1.03)	0.06	NS	NS
Previous sampling at another center	2.33 (0.89–6.07)	0.08	NS	NS
High‐volume centers	**1.62 (1.14–2.16)**	**0.02**	**2.12 (1.10–3.17)**	**0.03**
Neoplasm location (head or uncinate process)	0.93 (0.48–1.81)	0.84	–	–
Neoplasm size (mm)	1.03 (0.99–1.06)	0.08	**1.03 (1.00–1.06)**	**0.05**
rEUS‐FNB puncture route (transduodenal)	1.30 (0.69–2.42)	0.42	–	–
Tumor location (head)	0.80 (0.43–1.50)	0.49	–	–
Tumor location (uncinate)	1.65 (0.49–5.55)	0.42	–	–
Tumor location (body)	1.77 (0.77–4.08)	0.18	–	–
Tumor location (tail)	0.48 (0.20–1.15)	0.10	NS	NS
2nd generation FNB needles	1.52 (0.70–3.33)	0.29	–	–
rEUS‐FNB needle size				
25G	0.92 (0.44–1.93)	0.83	–	–
22G	1.03 (0.53–2.01)	0.92	–	–
20G	0.70 (0.20–2.47)	0.59	–	–
19G	2.20 (0.29–16.85)	0.45	–	–
rEUS‐FNB needle passes (no.)	1.24 (0.92–1.67)	0.15	–	–
rEUS‐FNB needle passes (>1)	0.95 (0.32–2.80)	0.93	–	–
rEUS‐FNB needle passes (>2)	1.75 (0.95–3.23)	0.07	NS	NS
rEUS‐FNB needle passes (>3)	1.13 (0.53–2.44)	0.75	–	–
ROSE availability	1.59 (0.72–3.52)	0.25	–	–
Type of sedation				
Conscious sedation	0.57 (0.31–1.06)	0.07	NS	NS
Deep sedation or general anesthesia	1.75 (0.95–3.23)	0.07	NS	NS

Variable significantly related to diagnostic accuracy on univariate or multivariate analysis are shown in bold.

CI, confidence interval; NS, not statistically significant; OR, odds ratio; ROSE, rapid on‐site evaluation.

On multivariate analysis, rEUS‐FNB performed at high‐volume centers (OR 2.12; 95% CI 1.10–3.17; *P* = 0.03), and larger lesions size (OR 1.03; 95% CI 1.00–1.06; *P* = 0.05), were identified as independently related to rEUS‐FNB diagnostic accuracy.

The specific performance of rEUS‐FNB in adequate cases (*n* = 435) is reported in Figure S1. In brief, patients in this group had a pretest probability (malignant disease prevalence) of 78% (72–83%). Of note, the probability of malignancy dropped to 17% after a negative rEUS‐FNB (12–26%), while it raised to 96% after a positive rEUS‐FNB (94–98%).

### Sample adequacy

rEUS‐FNB provided an adequate sample in 435 (94.2%, 95% CI 89.2–98.6%) cases (Table 2). Among the adequate samples, solid pancreatic lesions were considered malignant in 331 cases and benign in 104; in the remaining 27 cases, rEUS‐FNB samples were not adequate.

Among inadequate samples, 11 lesions were finally diagnosed as malignant and seven as benign, while the remaining nine cases could not be characterized according to the gold standards. The 2 × 2 results of true and false‐positive and ‐negative results are detailed in Table S3. ROC curve analysis identified lesion size >23 mm as the best cut‐off value for rEUS‐FNB sample adequacy (Fig. S2).

Factors related to rEUS‐FNB sample adequacy are reported in Table 4. In detail, on univariate analysis, rEUS‐FNB performed at high‐volume centers (OR 2.44; 95% CI 1.10–5.41; *P* = 0.03), lesion size >23 mm (OR 2.39; 95% CI 1.07–5.33; *P* = 0.03), and the use of second‐generation end‐cutting FNB needles (OR 4.42; 95% CI 1.94–10.08; *P* < 0.001) were directly related to sample adequacy. On multivariate analysis, the lesion size >23 mm (OR 3.04; 95% CI 1.31–7.06; *P* = 0.009) and the use of second‐generation end‐cutting FNB needles (OR 5.42; 95% CI 2.30–12.77; *P* < 0.001) were independently related to rEUS‐FNB sample adequacy.

**Table 4:** Factors related to repeated endoscopic ultrasound‐guided fine‐needle biopsy (rEUS‐FNB) sample adequacy

	Univariate	*P*‐value	Multivariate	*P*‐value
	OR (95% CI)		OR (95% CI)	
Gender (male)	0.67 (0.29–1.54)	0.340	–	–
Age (years)	0.99 (0.95–1.02)	0.720	–	–
Previous EUS‐FNB	1.03 (0.45–2.37)	0.940	–	–
Previous sampling at another center	1.45 (0.49–4.32)	0.490	–	–
High‐volume centers	**2.44 (1.10–5.41)**	**0.030**	NS	NS
Neoplasm location (head or uncinate process)	1.35 (0.60–3.05)	0.480	–	–
Neoplasm size (mm)	1.03 (0.99–1.07)	0.120		
Neoplasm size (>23 mm)	**2.39 (1.07–5.33)**	**0.030**	**3.04 (1.31–7.06)**	**0.009**
rEUS‐FNB puncture route (transduodenal)	1.22 (0.54–2.75)	0.640	–	–
Tumor location (head)	0.86 (0.38–1.95)	0.720	–	–
Tumor location (uncinate)	0.89 (0.14–13.6)	0.990	–	–
Tumor location (body)	0.83 (0.34–2.03)	0.680	–	–
Tumor location (tail)	0.69 (0.20–2.41)	0.570	–	–
2nd generation FNB needles	**4.42 (1.94–10.08)**	**<0.001**	**5.42 (2.30**–**12.77)**	**<0.001**
rEUS‐FNB needle size				
25G	0.70 (0.28–1.71)	0.440	–	–
22G	1.78 (0.80–3.99)	0.160	–	–
20G	0.35 (0.10–1.27)	0.150	–	–
19G	1.14 (0.15–8.86)	0.900	–	–
rEUS‐FNB needle passes	1.03 (0.72–1.48)	0.560	–	–
rEUS‐FNB needle passes (>1)	1.80 (0.59–5.48)	0.300	–	–
rEUS‐FNB needle passes (>2)	1.20 (0.54–2.69)	0.650	–	–
rEUS‐FNB needle passes (>3)	0.60 (0.25–1.43)	0.250	–	–
ROSE availability	1.07 (0.42–2.74)	0.890	–	–
Type of sedation				
Conscious sedation	1.53 (0.63–3.71)	0.340	–	–
Deep sedation or general anesthesia	0.65 (0.27–1.59)	0.340	–	–

Variable significantly related to sample adequacy on univariate or multivariate analysis are shown in bold.

CI, confidence interval; NS, not statistically significant; OR, odds ratio; ROSE, rapid on‐site evaluation.

## DISCUSSION

We demonstrated the optimal diagnostic yield of rEUS‐FNB in patients with pancreatic tumor after nondiagnostic or inconclusive results of previous EUS‐sampling. Indeed, FNB needles provided adequate samples in 95% of cases, leading to an accurate diagnosis in up to 90% of patients. To our knowledge, this is the first report on the performance of rEUS‐FNB, with a population that allows a reliable assessment of diagnostic yield and multivariate analysis for sample adequacy and diagnostic accuracy.

The analysis of the diagnostic accuracy was somewhat unexpected. Despite an optimal sensitivity (91.4%), we observed a low specificity (81.7%). In other words, after previous inconclusive results, there might be a substantial risk of rEUS‐FNB false‐positive results; this observation was confirmed by a recently published French article, reporting a 75% specificity in patients undergoing rEUS‐FNB.^15^ Among 12 patients with a false‐positive rEUS‐FNB pathology, most had mass‐forming chronic pancreatitis (*n* = 6) or an autoimmune pancreatitis (*n* = 2), in two cases normal pancreatic parenchyma was diagnosed, while the remaining two patients had a benign intrapancreatic benign lymph node and a pancreatic mycetoma (both confirmed by pathology on surgical specimen) (Table S3). Although the analysis of risk factors for false‐positive results was underpowered due to the low number of cases, it was shown that rEUS‐FNB at low‐volume centers, rEUS‐FNB at the same center as the first EUS‐guided tissue acquisition, tumors in the pancreatic head, and underlying chronic pancreatitis could be related to the reduced specificity. To reduce the risk of false‐positive results we suggest patients' referral to a high‐volume center for rEUS‐FNB, or at least a second pathology opinion in pancreatic head tumors, especially in the case of underlying chronic pancreatitis. While the EUS volume at each center was confirmed as an independent factor related to diagnostic accuracy, repeating EUS sampling in the same center could potentially imply an assessment bias by the same pathologist. Finally, it has been already shown that pancreatic head location and underlying chronic pancreatitis are potential limitations to diagnostic accuracy of EUS‐guided tissue acquisition.^16^, ^17^, ^18^

These results suggest that patients with solid pancreatic lesions should be referred to high‐volume centers after previous nondiagnostic or inconclusive EUS‐guided tissue acquisition, since a twofold increase in the final diagnostic accuracy can be expected. We speculate that this result can be explained not only by the increased endosonographer experience, but also by the skills of the entire team, including radiologists, surgeons, and pathologists.

The evaluation of the procedural details of the previous nondiagnostic or inconclusive EUS tissue sampling result confirmed several already demonstrated issues (Table S1). Most lesions were in the head or uncinate process, where EUS tissue sampling could be technically challenging and impaired by higher fibrosis. Interestingly, in 68.5% cases, one or two needle passes were performed. In those cases, up to 35% of tissue sampling was conducted with ROSE, which did not allow reaching a reliable result.

A recent meta‐analysis on rEUS‐FNA suggested that the ROSE presence was able to improve the diagnostic yield; however, in patients undergoing rEUS‐FNB, ROSE seemed to not be related to an improved diagnostic performance;^10^ this observation confirms the results of a recent large randomized controlled trial and a meta‐analysis that downsized the role of ROSE when second‐generation end‐cutting FNB needles are used.^19^, ^20^

The use of second‐generation end‐cutting FNB needles (i.e. Franseen‐type, fork‐tip, modified Menghini‐type with forward bevel, and Menghini‐type) was independently related to an increased sample adequacy rate, with a significant 5.42 OR (Tables 4, S4). In line with the main aim of this study and previous evidence,^10^ this result clearly demonstrates that, even in the case of a previous nondiagnostic or inconclusive finding, the use of FNB needles allows a better diagnostic outcome.

It was a thought‐provoking finding that a higher accuracy was observed in patients with previous FNA (93.6%) than FNB (87.9%). It is accepted that a small number of pancreatic tumors cannot be diagnosed through EUS‐sampling, independently from needle choice, techniques, the presence of ROSE, or operators' experience, mainly due to scant tumor cellularity with stromal proliferation in adenocarcinomas, or to cell atypia and severe fibrosis in pancreatitis. We speculated that it is more probable to reach an accurate diagnosis through an rEUS‐FNB in the case of previous EUS‐FNA, since more patients have been accurately characterized at the time of the first sampling when the EUS‐FNB needle was used; consequently, the relative number of cases that are “impossible” to be characterized through rEUS‐FNB is higher in those cases who underwent previous EUS‐FNB.

Finally, our study confirms that lesion size independently affects both rEUS‐FNB accuracy and adequacy, as demonstrated when FNA needles are used to characterize solid pancreatic lesions.^21^

The main limitation of our study is related to its retrospective design and the expected selection bias. In fact, the high prevalence of malignancy in this population (77%) suggests that a not‐negligible number of patients with benign conditions had not been referred for rEUS‐FNB. On the other hand, the focus of this research was not on the natural history of patients with inconclusive tissue sampling, but on the assessment of the diagnostic performance of FNB needles in this setting.

Another limitation is related to the gold standard adopted to evaluate the final diagnosis; in fact, we used strict parameters that did not allow drawing a definitive diagnosis in nine patients. These cases had an inadequate rEUS‐FNB sampling and a clinical/radiological follow‐up shorter than 12 months, with unavailable autopsy or proof of oncological disease. Nevertheless, we decided to include those patients in order to avoid underestimation of the sample adequacy and safety assessments. On the other hand, those cases have been censored from the diagnostic accuracy analysis, since no final diagnosis was reached according to the gold standard methods adopted.

Although the analysis of previous tissue sampling suggested several interesting considerations, those data were completely available in a proportion of the entire population (295 patients out of 462); therefore, a potential selection bias cannot be excluded, since most of them were available in those patients who underwent both first and repeated EUS‐FNB at the same center.

Finally, this study lacked a central pathology revision of rEUS‐FNB specimens; this issue could have contributed to the better outcomes in terms of both adequacy and accuracy observed in high‐volume centers. From a mere clinical point of view, we maintain that pathology revision of previous samples should be suggested together with patients' referral to high‐volume centers in this difficult clinical setting.

In conclusion, this study demonstrated the outstanding incremental diagnostic value of rEUS‐FNB after previous nondiagnostic or inconclusive EUS‐guided tissue acquisition. In detail, we demonstrated that patients' referral to high‐volume centers allowed a further increase in diagnostic yield, reducing the risk of false‐negative results. Finally, our results suggested that the use of second‐generation over standard FNB needles reduce the occurrence of inadequate samples, even in this potentially difficult patient population.

## CONFLICT OF INTEREST

Authors declare no conflict of interest for this article.

## FUNDING INFORMATION

None.

## Supporting information


**Appendix S1** Extensive description of the material and methods.

**Table S1** Technical and clinical characteristics of previous endoscopic ultrasound tissue acquisition.
**Table S2** Final diagnosis of solid pancreatic neoplasms based on gold standard methods.
**Table S3** Repeated endoscopic ultrasound‐guided fine‐needle biopsy results on solid pancreatic neoplasms with previous nondiagnostic or inconclusive results.
**Table S4** Detailed description of endoscopic ultrasound‐guided fine‐needle biopsy (EUS‐FNB) needles used for repeated EUS‐FNB.

**Figure S1** Nomogram reporting the diagnostic performance of repeated endoscopic ultrasound‐guided fine‐needle biopsy (rEUS‐FNB) in case of adequate tissue samples.

**Figure S2** Receiving operating characteristic (ROC) curve analysis for the identification of the best cut‐off point for lesion size related to repeated endoscopic ultrasound‐guided fine‐needle biopsy (rEUS‐FNB) sample adequacy.

## Data Availability

Raw data, analytic methods, and study materials will be made available to other researchers upon specific and motivated request.
