# Supplementary material for: Repeated endoscopic ultrasound‐guided fine‐needle biopsy of solid pancreatic lesions after previous nondiagnostic or inconclusive sampling
Source: Dig Endosc. 2023 Oct 25;36(5):615–24. doi: 10.1111/den.14686 (PMC12136261; doi:10.1111/den.14686)
Supplement: Supplementary file 1 — Appendix S1 Extensive description of the material and methods. [file DEN-36-615-s003.docx]

**Appendix S1 – Material and Methods**

**Methods**

*Study design.* Thirty Italian centers were invited to a retrospective study retrieving all consecutive patients who underwent rEUS-FNB for solid pancreatic lesions from January 2019 to December 2021. All adult patients (≥18-year-old) who underwent rEUS-FNB for solid pancreatic lesions characterization after previous non-diagnostic or inconclusive EUS-guided tissue acquisition were eligible.

Exclusion criteria were pancreatic cystic neoplasms, previous non-EUS guided pancreatic tissue sampling (trans-abdominal ultrasound or computed tomography), repeated tissue sampling different from EUS-FNB (EUS-fine needle aspiration, percutaneous, or surgical), and rEUS-FNB of extra-pancreatic neoplasms.

The study was firstly approved in December 2021 by our local Institution Review Board (IRB) (Comitato Etico di Area Vasta Emilia Centro, Italy, protocol number: 978-2021-OSS-AUSLIM-21185, ID 3369), and subsequently approved by all IRBs of each center. The study protocol was also made available on clinicaltrial.gov (NCT05226572).

All endoscopic procedures have been performed by physicians who have completed EUS training and have at least a 2-year experience and 150 EUS procedures/year; no trainees have been involved.

For each patient, the following variables were collected: age and gender, type of previous EUS tissue acquisition (either EUS-FNA or EUS-FNB), rEUS-FNB performed in the same center or after referral from another one, tumor location and size, needle size and design, number of needle passes, puncture route and presence of on-site pathologist for ROSE, type of sedation (conscious sedation, deep sedation or general anesthesia), results of rEUS-FNB (both the pathology report and the Bethesda classification), final diagnosis and type of gold-standard for diagnosis. All adverse events were recorded. Participating centers were divided into high or low volume centers based on the threshold of 150 EUS-FNB/year.

*Gold standard for diagnosis*. The gold standard for diagnosis of solid pancreatic lesions was: a) pathology of the surgical specimen for those who underwent pancreatectomy; b) pathology of autoptic examination in case of death, when available; c) at least 6-month assessment of disease evolution through a combination of clinical course, imaging modalities, and/or additional tissue sampling in non-resected patients with proven malignant disease; d) at least 12-month assessment through a combination of clinical course, imaging modalities, and/or additional tissue sampling demonstrating a stable benign condition.

In the remaining cases (i.e., inadequate rEUS-FNB sampling without confirmation or shorter follow-up) the patients were included in the rEUS-FNB sample adequacy analysis, but not in the assessment of diagnostic accuracy.

*Study aims and outcome definitions.* The primary end-point was to assess the diagnostic accuracy of rEUS-FNB of solid pancreatic lesions. Diagnostic accuracy was defined as the concordance between rEUS-FNB diagnosis and the gold standard diagnosis. Sample adequacy was defined as the acquisition through rEUS-FNB of a sufficient specimen to reach a pathology diagnosis. ^12^

The secondary end-points were rEUS-FNB sample adequacy, diagnostic sensitivity, specificity, positive and negative predictive values.

Procedure-related adverse events were defined as immediate or delayed (bleeding, perforation, pancreatitis, or any clinically relevant event) deemed as consequence of rEUS-FNB and were graded according to the AGREE classification. ^13,14^

End-cutting FNB needles, such as Franseen type, fork-tip, forward bevel, and Menghini type needles were included in the second-generation EUS-FNB group.

*Statistical analysis.* Continuous variables were expressed as mean ± standard deviation (SD) or median [interquartile range - IQR] according to their distribution and compared using the Student’s *t* test or the Mann-Whitney test, respectively. Categorical variables were reported as number and proportion and compared using the chi-square or Fisher’s exact tests, when appropriate. ROC curve analysis (Youden’s statistic) was used for dichotomization of continuous variables, when necessary.^15^

The logistic regression model was used to identify factors related to rEUS-FNB sample adequacy and diagnostic accuracy. Variables with a P value of <0.1 on univariate analysis were included in the multivariable logistic regression model. Odd ratios (OR) together with 95% confidence intervals (95% CI) have been reported. Statistical significance was determined as P<0.05 (two-tailed test). Statistical analyses were performed using MedCalc® Statistical Software version 20.110 (MedCalc Software Ltd, Ostend, Belgium; https://www.medcalc.org; 2022).
