# Supplementary material for: Repeated endoscopic ultrasound‐guided fine‐needle biopsy of solid pancreatic lesions after previous nondiagnostic or inconclusive sampling
Source: Dig Endosc. 2023 Oct 25;36(5):615–24. doi: 10.1111/den.14686 (PMC12136261; doi:10.1111/den.14686)
Supplement: Supplementary file 2 — Table S1 Technical and clinical characteristics of previous endoscopic ultrasound tissue acquisition. Table S2 Final diagnosis of solid pancreatic neoplasms based on gold standard methods. Table S3 Repeated endoscopic ultrasound‐guided fine‐needle biopsy results on solid pancreatic neoplasms with previous nondiagnostic or inconclusive results. Table S4 Detailed description of endoscopic ultrasound‐guided fine‐needle biopsy (EUS‐FNB) needles used for repeated EUS‐FNB. [file DEN-36-615-s004.docx]

**Supplementary Table 1.** Technical and clinical characteristics of previous endoscopic ultrasound tissue acquisition

|  | **Total**  (*no.* 295) |
| --- | --- |
| **Demographic** |  |
| Gender (male), *no. (%)* | 136 (46.1%) |
| Age (years), *median [IQR]* | 73 [58 – 79] |
| **Solid pancreatic neoplasms** |  |
| Location |  |
| Head, *no. (%)* | 186 (63.1%) |
| Uncinate process, *no. (%)* | 24 (8.1%) |
| Body, *no. (%)* | 56 (19.0%) |
| Tail, *no. (%)* | 29 (9.8%) |
| Size (mm*), median [IQR]* | 23 [19-36] |
| **Previous EUS-TA details** |  |
| Needle used |  |
| EUS-FNA needle, *no. (%)* | 79 (26.8%) |
| EUS-FNB needle, *no. (%)* | 216 (63.2%) |
| At the same center, *no. (%)* | 270 (91.5%) |
| At another center, *no. (%)* | 25 (8.5%) |
| High-volume center, *no. (%)* | 207 (70.2%) |
| Puncture route |  |
| Trans-gastric, *no. (%)* | 90 (30.5%) |
| Trans-duodenal, *no. (%)* | 205 (69.5%) |
| Needle size |  |
| 25 gauge, *no. (%)* | 105 (35.6%) |
| 22 gauge, *no. (%)* | 158 (53.6%) |
| 20 gauge, *no. (%)* | 12 (4.1%) |
| 19 gauge, *no. (%)* | 20 (6.8%) |
| Needle passes |  |
| 1 pass, *no. (%)* | 46 (15.6%) |
| 2 passes, *no. (%)* | 156 (52.9%) |
| 3 or more passes, *no. (%)* | 93 (31.5%) |
| ROSE availability, *no. (%)* | 104 (35.3%) |

Abbreviations: EUS: endoscopic ultrasound; EUS-FNA: EUS-fine needle aspiration; EUS-FNB: EUS-fine needle biopsy; EUS-TA: EUS-tissue acquisition; IQR: interquartile range; ROSE: rapid-on-site-evaluation.

**Supplementary table 2.** Final diagnosis of solid pancreatic neoplasms based on gold standards methods

| **Final diagnosis** | **Total**  (*no.* 462) |
| --- | --- |
| **Malignant conditions** | No. 349 (75.5%) |
| Pancreatic adenocarcinoma, *no. (%)* | 267 |
| Neuroendocrine tumor, *no. (%)* | 48 |
| Metastases, *no. (%)* | 22 |
| Neuroendocrine carcinoma, *no. (%)* | 6 |
| Solid pseudopapillary tumor, *no. (%)* | 2 |
| Sarcoma, *no. (%)* | 2 |
| Lymphoma, *no. (%)* | 1 |
| Gastrointestinal stromal tumor, *no. (%)* | 1 |
| **Benign conditions** | No. 104 (22.5%) |
| Chronic pancreatitis, *no. (%)* | 49 |
| Normal pancreatic parenchyma, *no. (%)* | 28 |
| Autoimmune pancreatitis, *no. (%)* | 18 |
| Benign lymphoid tissue, *no. (%)* | 5 |
| Accessory spleen, *no. (%)* | 2 |
| Micetoma, *no. (%)* | 1 |
| Ganglioneuroma, *no. (%)* | 1 |
| **Not characterized*** | No. 9 (1.9%) |
| Undetermined, *no. (%)* | 9 |

*9 cases could not be characterized with the gold standard methods adopted

Abbreviations: EUS: endoscopic ultrasound; EUS-FNA: EUS-fine needle aspiration; EUS-FNB: EUS-fine needle biopsy; EUS-TA: EUS-tissue acquisition; IQR: interquartile range; ROSE: rapid-on-site-evaluation.

**Supplementary table 3.** Repeated EUS-fine needle biopsy results on solid pancreatic neoplasms with previous non-diagnostic or inconclusive results.

| **Diagnosis** | **Total**  (*no.* 462) | **True**  **positive** | **False**  **negative** | **False**  **positive** | **True negative** | **Not adequate**** |
| --- | --- | --- | --- | --- | --- | --- |
| **Malignant conditions** | No. 349 (75.5%) | 319 | 19 | --- | --- | 11 |
| PC | 267 | 243 | 17 | --- | --- | 7 |
| NET | 48 | 45 | 0 | --- | --- | 3 |
| Metastases | 22 | 20 | 1 | --- | --- | 1 |
| NEC | 6 | 5 | 1 | --- | --- | 0 |
| SPT | 2 | 2 | 0 | --- | --- | 0 |
| Sarcoma | 2 | 2 | 0 | --- | --- | 0 |
| Lymphoma | 1 | 1 | 0 | --- | --- | 0 |
| GIST | 1 | 1 | 0 | --- | --- | 0 |
| **Benign conditions** | No. 104 (22.5%) | --- | --- | 12 | 85 | 7 |
| CP | 49 | --- | --- | 6 | 40 | 3 |
| Normal pancreas | 28 | --- | --- | 2 | 24 | 2 |
| AIP | 18 | --- | --- | 2 | 14 | 2 |
| Benign LN | 5 | --- | --- | 1 | 4 | 0 |
| Accessory Spleen | 2 | --- | --- | 0 | 2 | 0 |
| Micetoma | 1 | --- | --- | 1 | 0 | 0 |
| Ganglioneuroma | 1 | --- | --- | 0 | 1 | 0 |
| **Not characterized*** | No. 9 (1.9%) | N/A | N/A | N/A | N/A | 27 |

*9 cases could not be characterized with the gold standard methods adopted.

** repeated EUS-fine needle biopsy sample was not adequate.

Abbreviations: PC: pancreatic cancer; NET: neuroendocrine tumor; NEC: neuroendocrine cancer; SPT: solid pseudopapillary tumor; GIST: gastrointestinal stromal tumor; CP: chronic pancreatitis; AIP: autoimmune pancreatitis; LN: lymph node.

**Supplementary Table 4**. Detailed description of endoscopic ultrasound fine-needle biopsy (EUS-FNB) needle used for repeated EUS-FNB.

| **Needle type** | **Second-generation EUS-FNB needle** | **Needle name** | **Manufacturer** | **Needle size** | **Number of cases each needle was used** |
| --- | --- | --- | --- | --- | --- |
| Reverse bevel | No | EchoTip ProCore | Cook Endoscopy | 25 gauge  22 gauge  19 gauge | No. 16  No. 28  No. 4 |
| Antegrade bevel | Yes | EchoTip ProCore | Cook Endoscopy | 20 gauge | No. 22 |
| Franseen type | Yes | Acquire | Boston Scientific Corp. | 25 gauge  22 gauge  19 gauge | No. 44  No. 170  No. 1 |
| Franseen type | Yes | SonoTip TopGain | Medi-Globe | 25 gauge | No. 1 |
| Franseen type | Yes | Trident | Micro-Tech Endoscopy | 19 gauge | No. 4 |
| Fork-tip | Yes | SharkCore | Medtronic | 25 gauge  22 gauge  19 gauge | No. 34  No. 109  No. 4 |
| Menghini | Yes | EZ-Shot 3 Plus | Olympus Corp. | 25 gauge  22 gauge  19 gauge | No. 1  No. 17  No. 7 |
