# Supplementary figures and images for: Repeated endoscopic ultrasound‐guided fine‐needle biopsy of solid pancreatic lesions after previous nondiagnostic or inconclusive sampling
Source: Dig Endosc. 2023 Oct 25;36(5):615–24. doi: 10.1111/den.14686 (PMC12136261; doi:10.1111/den.14686)

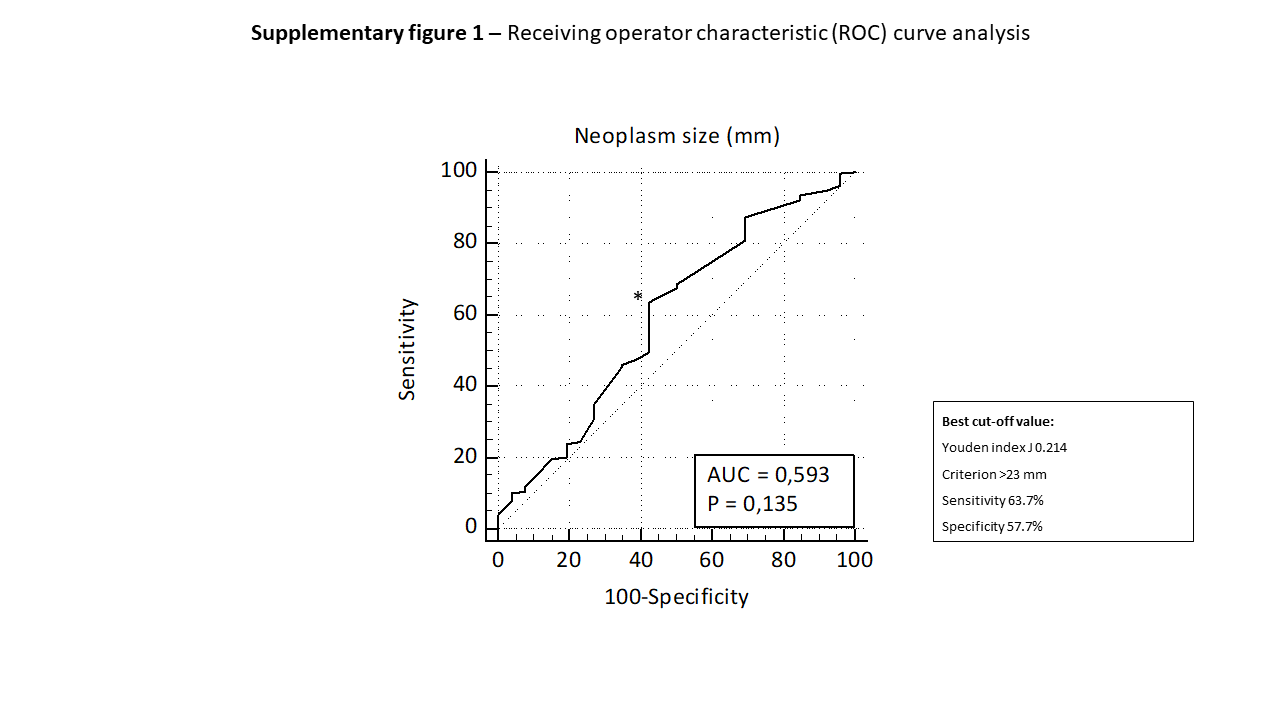

Supplement: Supplementary file 3 — Figure S1 Nomogram reporting the diagnostic performance of repeated endoscopic ultrasound‐guided fine‐needle biopsy (rEUS‐FNB) in case of adequate tissue samples. [file DEN-36-615-s001.tif]

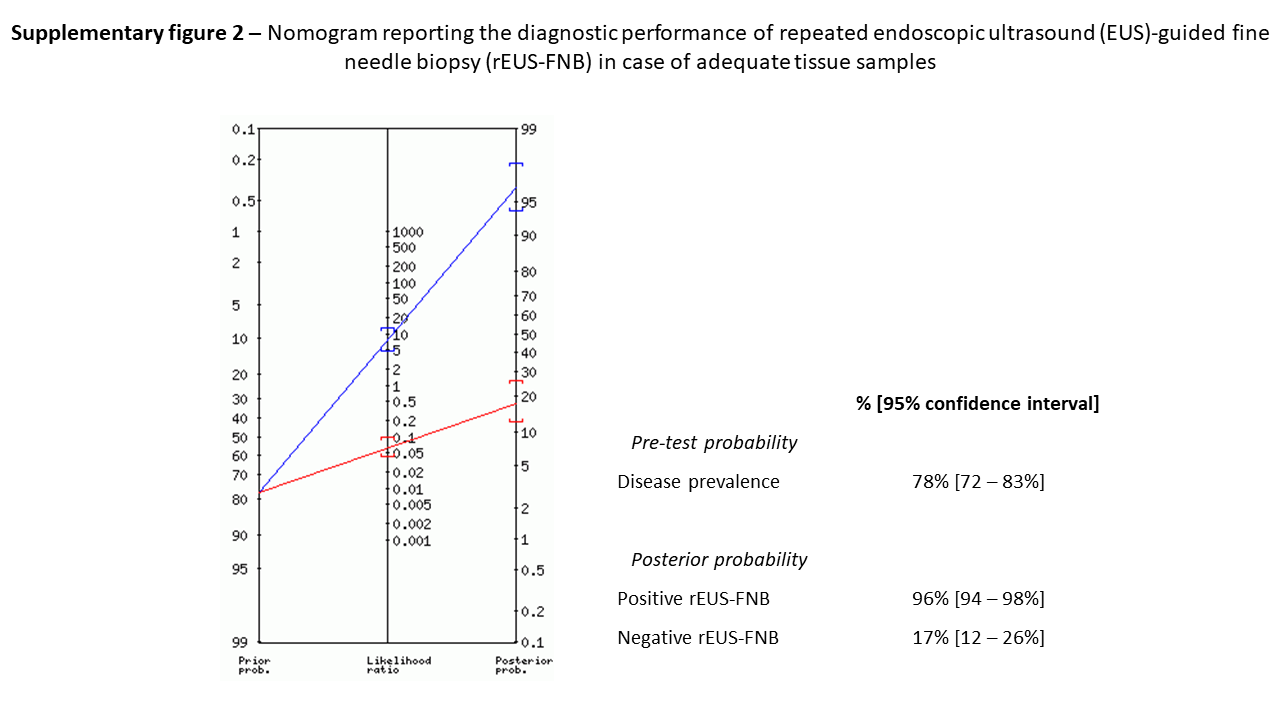

Supplement: Supplementary file 4 — Figure S2 Receiving operating characteristic (ROC) curve analysis for the identification of the best cut‐off point for lesion size related to repeated endoscopic ultrasound‐guided fine‐needle biopsy (rEUS‐FNB) sample adequacy. [file DEN-36-615-s002.tif]
